# Enhancing Mechanical Behavior and Energy Dissipation in Fiber-Reinforced Polymers through Shape Memory Alloy Integration: A Numerical Study on SMA-FRP Composites under Cyclic Tensile Loading

**DOI:** 10.3390/ma16165695

**Published:** 2023-08-19

**Authors:** Saeed Eilbeigi, Mohammadreza Tavakkolizadeh, Amir R. Masoodi

**Affiliations:** Department of Civil Engineering, Ferdowsi University of Mashhad, Mashhad 9177948974, Iran; saeed_eilbeigighalani@mail.um.a.ir

**Keywords:** shape memory alloy, SMA-FRP composite, finite element method, tensile properties, cyclic loading

## Abstract

Conventional fiber-reinforced polymers (FRPs) have a relatively linear stress–strain behavior up to the failure point. Therefore, they show brittle behavior until the failure point. Shape memory alloys, in addition to having high ductility and good energy dissipation capability, are highly resistant to corrosion and show good performance against fatigue. Therefore, using the SMA fibers in the production of FRPs can be a suitable solution to solve the problem of the brittle behavior of conventional FRPs. SMA fibers can be integrated with a polymeric matrix with or without conventional fibers and create a new material called SMA-FRP. This study investigates the effect of using different volume fractions of conventional fibers (carbon, glass, and aramid) and SMA fibers (NiTi) in the super-elastic phase and the effect of the initial strain of SMA fibers on the behavior of SMA-FRP composites under cyclic tensile loading. Specimens are designed to reach a target elastic modulus and are modeled using OpenSees (v. 3.5.0) finite element software. Analyzing the results shows that in the SMA-FRP composites that are designed to reach a target elastic modulus, with an increase in the volume fraction of SMA fibers, the maximum stress, residual strain, and strain hardening ratio are reduced, and the ability to energy dissipation capability and residual stress increases. It was also observed that increasing the percentage of the initial strain of SMA fibers increases the maximum stress and energy dissipation capability and reduces the residual strain and yield stress. In the investigation of the effect of the type of conventional fibers used in the construction of composites, it was found that the use of fibers that have a larger failure strain increases the maximum stress and energy dissipation capability of the composite and reduces the strain hardening ratio. In addition, increasing the elastic modulus of conventional fibers increases the residual strain and residual stress of the composites.

## 1. Introduction

Smart systems can adapt themselves to different loading conditions [1,2,3,4]. In recent years, using lighter, stronger, and smarter materials has increased in different industries, including aerospace, military, transportation, and the construction of different structures [5]. Because of the large strength-to-weight ratio, high corrosion resistance, and suitable durability, fiber-reinforced polymer (FRP) composites are widely used in engineering applications [6]. Some studies have been conducted on the application of hybrid nanocomposites in different engineering sciences [7,8]. However, conventional FRPs, such as carbon-fiber-reinforced polymer (CFRP), glass-fiber-reinforced polymer (GFRP), and aramid-fiber-reinforced polymer (AFRP), have a relatively linear stress–strain behavior up to the failure point. Therefore, they show brittle behavior at the failure point. Thus, they have less energy dissipation capability and perform poorly in structures requiring high ductility [9,10]. One of the materials used in smart systems in recent years is shape memory alloys (SMAs) [3,11]. SMAs are a group of metal alloys that can recover large values of strains during unloading. SMAs are highly resistant to corrosion and show good performance against fatigue. SMAs have good energy dissipation capability because loading and unloading paths do not match each other and create hysteresis cycles. This exclusive property of SMAs has made them a suitable choice for developing smart materials [12,13].

The crystalline structure of a material specifies the material’s behavior. As such, it is important to have a basic understanding of the microscopic aspects of SMAs. SMAs can be found in one of two stable phases, martensitic or austenitic. Austenite occurs at high temperatures and its crystal structure has a high order of symmetry. The martensitic phase is stable in either a multi-variant twinned form or a single variant detwinned form where the multi-variants represent a change in the orientation of the crystalline structure [14]. For NiTi (used in this study), the crystal structure of the austenite phase is ‘Body Centered Cubic’.

SMAs have two unique characteristics: shape memory effect (SME) and super-elasticity (SE). In the shape memory effect, deformations fall back to their original un-deformed shape through heating, whereas in the super-elasticity characteristic, the deformations are recovered after unloading. Both characteristics depend on the material temperature. SMA is in the austenitic phase at relatively high temperatures (more than A_f_), and consequently shows super-elastic behavior. However, the SMA is in a martensitic phase at relatively low temperatures (lower than M_f_) and shows shape memory effect behavior [10,14].

**Figure 1:**
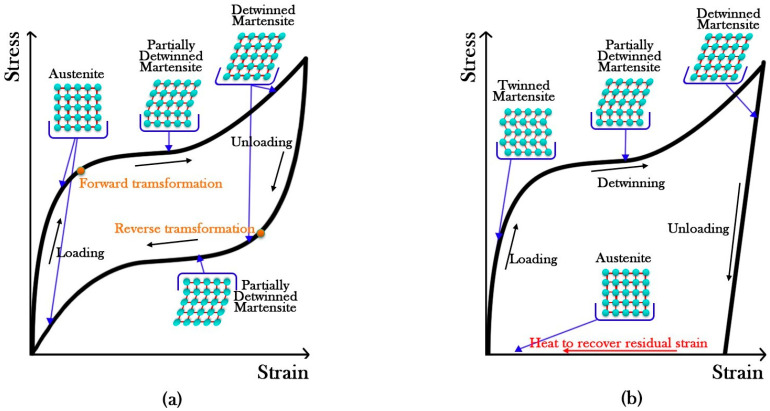
Schematic of the stress–strain curve of SMA (**a**) super-elastic behavior (T > A_f_) (**b**) shape memory effect (T < M_f_).

Figure 1 illustrates the shape memory effect and super-elastic behavior in SMAs. SMAs are applied in different fields such as biomedical, aerospace, and civil structures [15]. In civil structures, different shapes of SMAs are used for the retrofitting and strengthening of structures, such as application in seismic isolators [16,17,18,19], steel beam–column connections [20,21,22], bracing systems [23,24,25,26,27], different types of dampers [28,29,30,31], reinforced concrete beams [32,33,34], reinforced concrete columns [35,36,37,38,39,40,41], reinforced concrete slabs [42], reinforced concrete beam–column connections [43,44,45,46], and wood structures [47,48].

**Figure 2:**
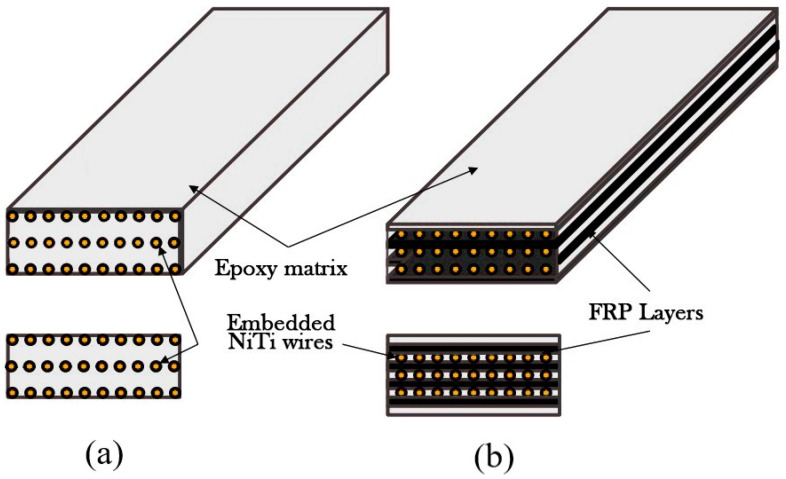
Schematic of SMA-FRP composites: (**a**) pure composite, (**b**) hybrid composite.

Many researchers have investigated the application of SMA wires as fibers in epoxies. Shape memory alloy–fiber-reinforced polymer (SMA-FRP) composite is a heterogeneous material type that is composed of a polymeric matrix material reinforced with SMAs with or without supplementary conventional fibers. Resins are the bonding component of reinforcement fibers and connect SMA fibers and other conventional fibers (e.g., glass, carbon, and aramid) integrally [14]. Figure 2 shows a schematic of an SMA-FRP composite.

Jang et al. [49] concluded that embedding nitinol wires in CFRP composites decreases their tensile strength from 1300 MPa to 1100 MPa, and the tensile strength can be reduced up to 1050 MPa regarding the orientation of CFRP layers and nitinol wires. The reason for this reduction is that the embedding of nitinol wires in CFRP composites causes deficiencies in materials and decreases their tensile strength. One of the other studies on SMA composites leading to poor results was conducted by Xu et al. [50]. This weakness is attributed to insufficient interfacial bonding of SMA wires and resin because of air voids around SMA wires in the composite. In another study by Xu et al. [51], SMA wires with small diameters were employed to improve the interfacial bonding of hybrid composites with the study of a composite reinforced with ultrathin SMA wires and carbon fibers. Regarding the results of these studies, it was observed that SMA wires with an ultrathin diameter almost eliminate the voids adjacent to the SMA wires when conventional reinforcement in the composite was directed parallel to the SMA wires and greatly reduced voids when the reinforcement was directed perpendicular to them. In the continuation of studies conducted on SMA-FRP composites, some methods were examined for producing SMA-FRP composites by different researchers [52,53,54]. In another study, Wierschem and Andrawes [55] utilized SMA wires and glass fibers in GFRP composites and showed that SMA wires could improve the ductility of composites. Nissle et al. [56] used super-elastic SMA wires with conventional FRP composites to avoid damage propagation in composites. Sharifi et al. [57] produced a smart FRP composite consisting of two layers. A layer was reinforced with unidirectional carbon fibers, and SMA wires reinforced another layer. In this investigation, asymmetric specimens showed very low tensile strength. Pappada et al. [54], Nissle et al. [56], Cohades et al. [58], and Lidan Xu et al. [59] used super-elastic SMA wires with conventional FRP composites to increase their impact resistance and avoid damage propagation in composites. Zafar and Andrawes [10,14] produced SMA-FRP composites using SMA and glass fibers and tested them under uniaxial tensile loading. The results indicate that SMA-FRP composites recover the generated strains with minimal residual deformation.

In addition to using SMA wires with conventional fibers in manufacturing composites, the use of SMA wires as the only reinforcing fibers in SMA-FRP composites has also been investigated. Wierschem [60] conducted an experimental study on SMA-FRP composites. Although the percentage of SMA fibers used in composites was low, the results indicated that SMA-FRP composites show high ductility. Payandeh et al. [61] investigated the effect of SMA wires on composites’ behavior. They examined some composites with nitinol wires at different temperatures under unidirectional tensile loading. Zafar and Andrawes [10] manufactured a specimen of SMA-FRP composite with SMA fibers and tested it under uniaxial tensile loading. Results indicated that SMA-FRP composites could recover the strains with minimal residual deformation. Dagash et al. [9] examined the cyclic behavior of composites produced with SMA wires. Results indicated that increasing the volume fraction of SMA wires in composites that are reinforced using only SMA wires, minimizes the residual strains and increases energy dissipation capability. Saeedi and Shokrieh [62] investigated the effect of SMA wires in composites on damage propagation reduction and increasing strength against crack growth. Studies by Dagash et al. [9] and Wang et al. [63] indicated that SMA wires increase the composites’ fatigue strength.

This study investigates the effect of using different volume fractions of conventional fibers (carbon, glass, and aramid) and SMA fibers (NiTi) in the super-elastic phase and the effect of the initial strain of SMA fibers on the behavior of SMA-FRP composites under cyclic tensile loading. In this study, the SMA properties, properties of each conventional fiber (carbon, glass, and aramid), matrix properties, and cross-sectional area of composites are considered constant in all specimens. The reinforcing fibers were selected to cover a wide range of elastic modulus and failure strain [60]. The innovation and strength of this investigation lies in its systematic approach to evaluating various factors that influence the mechanical properties of SMA-FRP composites. The study’s focus on target elastic modulus as a design parameter is relevant for engineering applications. Furthermore, investigating the impact of different types of conventional fibers provides insights into material selection.

## 2. Specimens Introduction

Different alloys can be classified as shape memory alloys. However, most research has focused on an alloy composed of almost the same fraction of nickel and titanium. Ni-Ti SMAs, known as nitinol, were introduced by Buehler and Wiley in 1965. Buehler and Wiley discovered this alloy in their experiments in the Naval Ordnance Laboratory [64]. Nitinol is the most conventional SMA used in structural applications due to its excellent super-elastic properties, lower sensitivity to temperature variation, high corrosion resistance, and high fatigue resistance [65]. The point that should be considered is that although copper- and iron-based alloys are more economical than nitinol alloys, their thermal and mechanical properties are less desirable than nitinol. Therefore, nitinol is the most popular and widely used shape memory alloy [66].

Specimens are designed to reach a target elastic modulus. The specimens are subjected to cyclic tensile loading. After determining the stress–strain curve of each specimen under the applied load, the results are discussed in terms of the maximum stress, yield stress, residual stress, residual strain, energy dissipation capability, and strain hardening ratio. Table 1 presents the mechanical properties of materials used in this research. Nitinol wires with a diameter of 0.5 mm were used to produce SMA-FRP composites. In addition, the cross-sectional area of all composites is assumed to be 6.77 mm^2^.

As mentioned, this study investigates the effect of using different fiber volume fractions (carbon, glass, aramid, and nitinol) and the effect of the initial strain of SMA fibers on the SMA-FRP composites’ behavior under cyclic tensile loading. For investigating the effect of conventional fiber types on the SMA-FRP composite behavior, the composites are divided into six groups. These groups are introduced in Table 2.

In the first group, three composites containing only SMA fibers with different volume fractions are investigated to study the effect of SMA fiber volume fraction on the behavior of SMA-FRP composites. In other groups, since the volume fraction of SMA fibers and conventional fibers in each group significantly affects the composite mechanical properties, composites must be designed to reach a specific characteristic [14]. The design of hybrid SMA-FRP composites to achieve a target elastic modulus (E_c_) is conducted by changing the volume fraction of conventional reinforcing fibers and SMA fibers. For this purpose, the S7 composite with 20% SMA fibers is utilized as a benchmark in terms of setting a target stiffness of hybrid composites in the G2 to G6 groups. The rule of mixture (ROM) is the simplest model to predict the elastic properties of composite materials reinforced with unidirectional continuous fibers [68]. Since the fibers are parallel to the direction of the load application, Equation (1) shows the relationship between volume fraction and elastic modulus of fibers, resin, and composite [14]:

(1) $$E_{c}=\left(E_{f}\right)\left(V_{f}\right)+\left(E_{m}\right)\left(V_{m}\right)+(E_{SMA})(V_{SMA})$$

For a given SMA fiber volume fraction, the conventional fiber volume fraction is calculated as Equation (2) [14]:

(2) $$V_{f}=\frac{E_{c}-\left(E_{m}\right)\left(V_{m}\right)-(E_{SMA})(V_{SMA})}{E_{f}}$$

In these equations, $E_{c}$, $E_{f}$, $E_{m}$, and $E_{SMA}$ are the elastic modulus of composite, conventional fibers, resin, and SMA fibers, respectively. In addition, $V_{f}$, $V_{m}$, and $V_{SMA}$ are the volume fraction of conventional fibers, resin, and SMA fibers. In addition, one of the important parameters that can affect the behavior of composites made of SMA fibers is the initial strain in the SMA fibers used in producing SMA-FRP composites [60]. In this study, the initial strain of SMA fibers for producing different specimens is assumed to be 0, 0.25%, and 0.5%. The initial strain of SMA fibers is not considered a higher value because according to the mechanical properties of SMA used in this study (Table 1) and its flag-shaped stress–strain curve, the SMA phase is converted from austenitic to martensitic at the strain of 0.61%. The initial strain is selected so that the SMA fibers do not undergo a phase transformation from austenitic to martensitic and do not lose their stiffness before loading the composite.

Table 3 shows 48 SMA-FRP composites with different types of conventional fibers, different volume fractions of SMA fibers, different volume fractions of conventional fibers, and different values of the initial strain of SMA fibers. The rules for the naming of test specimens are as follows: The letters before the first dash represent the type of conventional fibers. LC is used for low-modulus carbon fibers, HC is used for high-modulus carbon fibers, SG is used for S-Glass fibers, EG is used for E-Glass fibers, and AR is used for aramid fibers. After the first dash, the letter S is used along with a number that indicates the number of SMA fibers that are used in producing the composites. Finally, the number after the second dash refers to the initial strain level of SMA fibers (00, 25, and 50 are used for 0%, 0.25%, and 0.50%, respectively). It is noted that for G1 group specimens that are made only from SMA fibers, naming the sample is done using the letter S along with a number that indicates the number of SMA fibers.

## 3. Specimen Numerical Modeling

**Figure 3:**
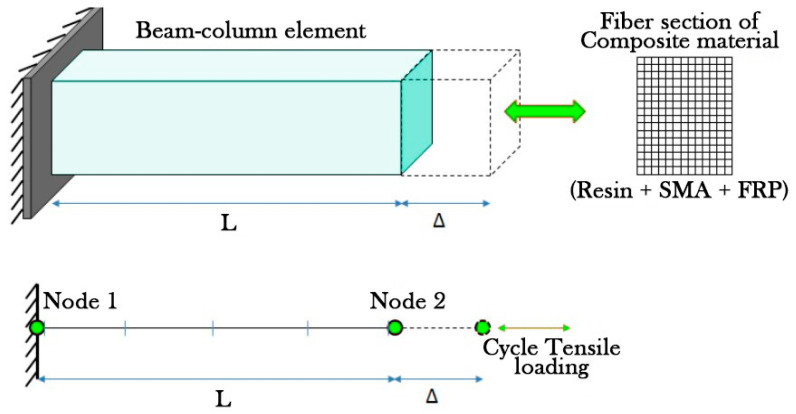
Beam-column element with boundary conditions and the fiber section approach.

The finite element software, OpenSees, was utilized to develop numerical models. To model the composites, a simple cantilever beam was considered using the nonlinear beam-column element in the OpenSees library. This element is based on iterative force formulation and considers the spread of plasticity behavior along the element length. The integration along the element is based on the Gauss–Lobatto quadrature rule (two integration points at the element ends). The fiber-section model is used for obtaining the stress–strain curves of examined composites. In this method, the area of the cross-section is divided into finite regions, and the behavior of constituent materials of the SMA-FRP composite (epoxy, FRP, SMA) is assigned to each region [14]. Figure 3 shows the beam-column elements, the boundary conditions, and the fiber section approach. As seen, a simple cantilever beam with a cross-section was modeled with two nodes. Node 1 was considered fixed for all three DOF, while node 2 was free for all three DOF. The element with length (L) underwent cyclic tensile elongation at node 2 [14].

**Figure 4:**
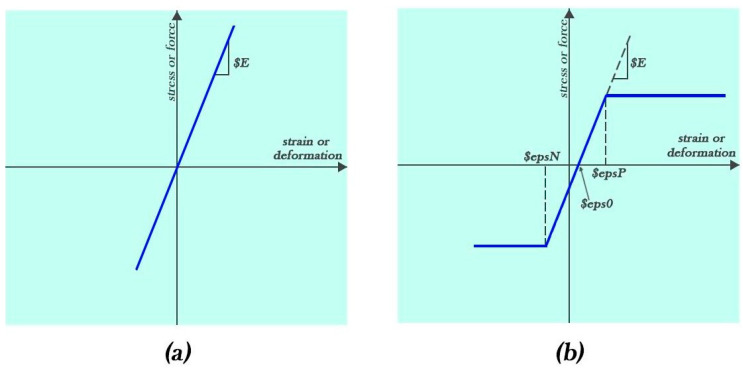
Stress–strain curve of (**a**) conventional fiber and (**b**) resin.

A linear elastic material model is used for modeling the conventional fibers (glass, carbon, and aramid) in OpenSees. The stress–strain curve of this material is indicated in Figure 4a [69]. The elastic perfectly plastic (EPP) material model is used to model the resin, and its stress–strain curve is shown in Figure 4b [69].

**Figure 5:**
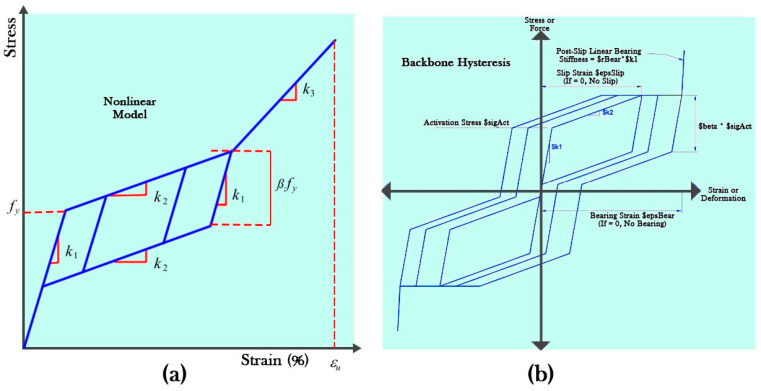
Stress–strain curve of (**a**) SMA materials and (**b**) self-centering material.

The self-centering material model is used to model SMA materials in OpenSees. The data required for defining the flag-shaped stress–strain curve of SMA materials (Figure 5a) [70] should be converted to data required for defining the stress–strain curve of self-centering materials (Figure 5b) [69].

In this study, the specimens are subjected to cyclic tensile loading. The loading protocol applied to composites is strain control. The strain value is increased by 0.5% for each loading and unloading cycle. This trend is continued until the strain becomes 4%. In addition, the loading and unloading cycles are applied so that the specimen is not affected by compression stresses [10,14].

**Figure 6:**
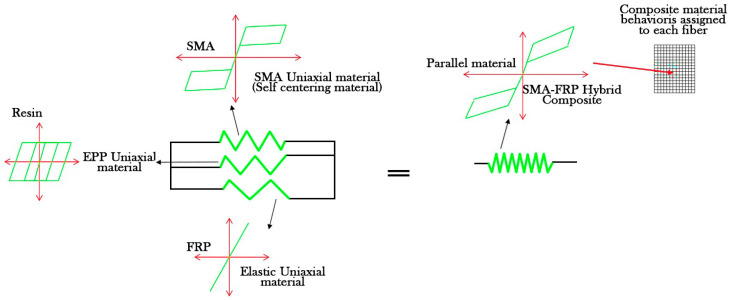
Parallel materials used for combining resin, SMA fibers, and conventional fibers.

The parallel material command was utilized for joining the resin, SMA fibers, and conventional fibers in SMA-FRP composite modeling via OpenSees software. In the parallel command, the strains are equal across all composite materials constituents, whereas the stress and stiffness values are accumulated together. The schematic of parallel material used for modeling SMA-FRP composites is presented in Figure 6.

Numerical simulation in OpenSees software should be calibrated with experimental results. The PRC3 experimental specimen from Zafar et al.’s research [14] is utilized for calibrating the numerical simulation. In Zafar et al.’s research, which was used to investigate the compatibility of finite element modeling with experimental results, the coupon specimens made out of hybrid configurations were termed as ‘Partially Reinforced Composite’ (PRC). PRC-3 is a Partially Reinforced Composite with 3 SMA wires.

The Mechanical properties of constituents of the PRC3 composite are listed in Table 1. In addition, the characteristics of the experimental specimen are presented in Table 4.

**Figure 7:**
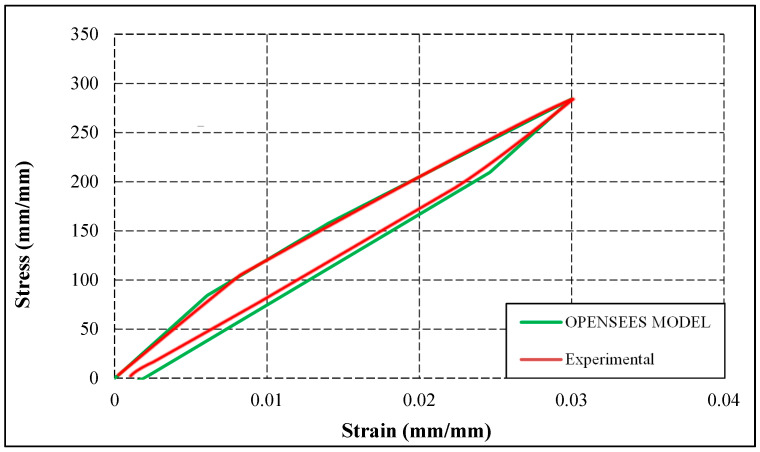
Comparison of stress–strain curves of experimental results and numerical simulation results.

As mentioned, OpenSees software was utilized to develop numerical models. A linear elastic material model, elastic perfectly plastic (EPP) material model, and self-centering material model have been used to introduce the behavior of glass fibers, resin, and SMA fibers, respectively. In addition, a parallel springs model has been used to join these materials. After modeling the specimen with OpenSees software, cyclic loading is applied to the specimen, and the stress–strain curve of the numerical model is analyzed and compared with the stress–strain curve obtained from the experimental study. Figure 7 shows the stress–strain curves of the experimental study and numerical simulation in OpenSees and a comparison between numerical and experimental results for the PRC3 composite. Results indicate that the numerical simulation used in this study can suitably predict the initial stiffness, stresses, transformation strains (phase change), and post-yield behavior.

## 4. Results and Discussion

**Figure 8:**
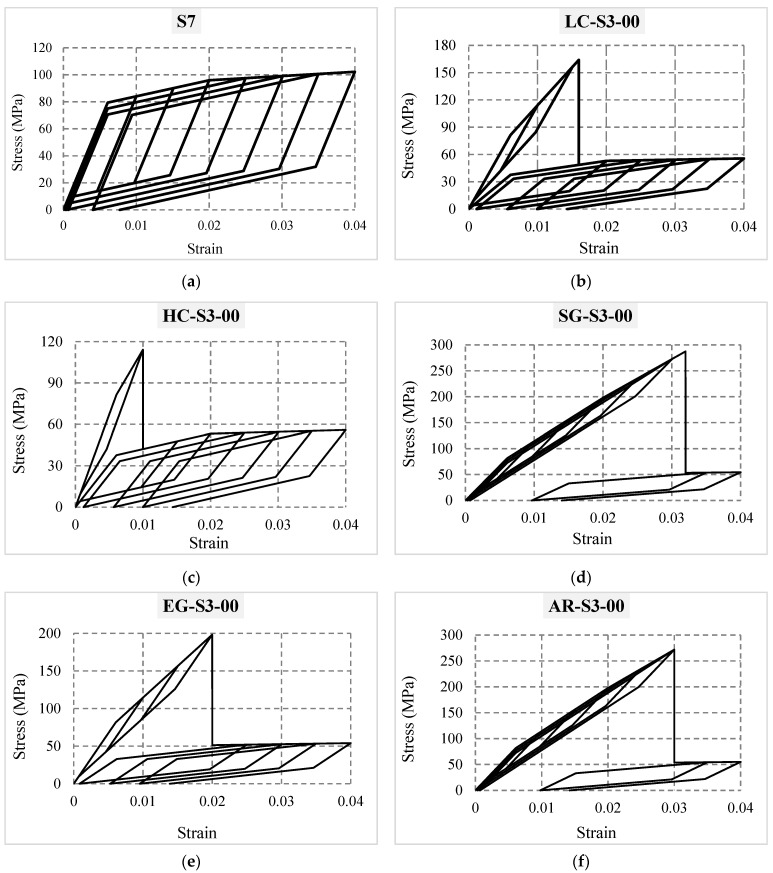
Stress–strain curves of some composites in groups G1 to G6: (**a**) S7 composite; (**b**) LC-S3-00 composite; (**c**) HC-S3-00 composite; (**d**) SG-S3-00 composite; (**e**) EG-S3-00 composite; (**f**) AR-S3-00 composite.

After calibrating the numerical simulation results obtained from OpenSees software, specimens introduced in Table 3 were modeled in OpenSees and subjected to the introduced loading protocol. The hysteresis stress–strain curves of some composites in groups G1 to G6 are shown in Figure 8 (one curve is presented from each group to avoid repetition).

After modeling and analysis of studied specimens, obtained results are presented using tables and curves. The results are presented in terms of the maximum stress, yield stress, residual stress, residual strain, energy dissipation capability, and strain hardening ratio.

### 4.1. Maximum Stress

**Figure 9:**
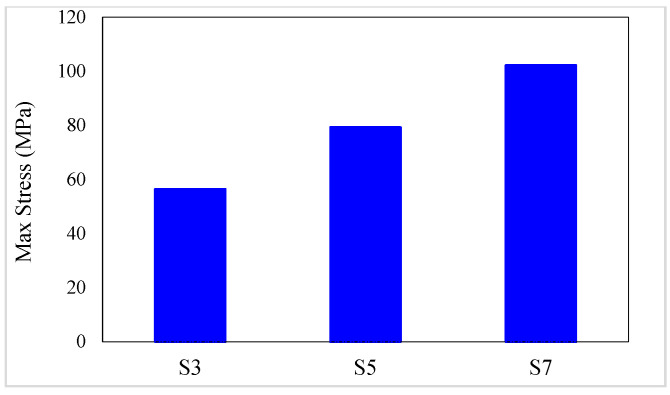
Variation of maximum stress in G1 group composites.

**Figure 10:**
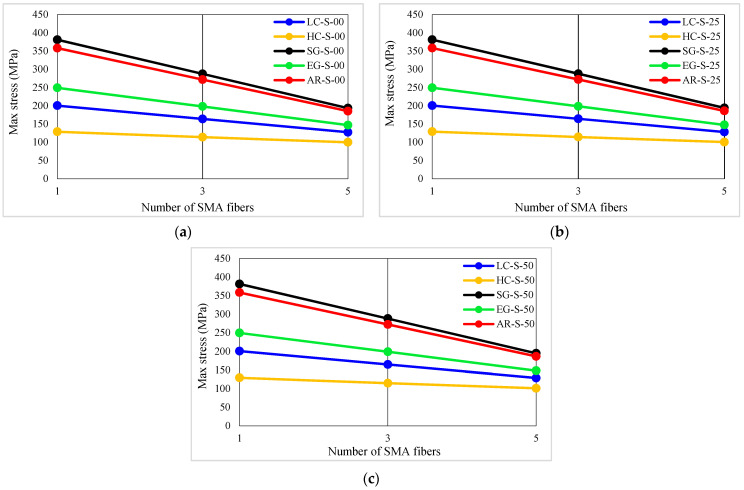
Variation of maximum stress in G2 to G6 group composites: (**a**) pre-strain = 0%, (**b**) pre-strain = 0.25%, (**c**) pre-strain = 0.50%.

The maximum stress values of studied specimens are presented in Table 5. The maximum stress is equal to the stress at the peak point of the stress–strain curves of composites. The pick point of the stress–strain curves is the conventional fiber failure point. In addition, the maximum stress variation diagrams versus the conventional fiber type, the volume fraction of conventional and SMA fibers, and the initial strain of SMA fibers are shown in Figure 9 and Figure 10. By considering Table 5 and plotted diagrams in Figure 9 and Figure 10, the following results were concluded:

- Evaluating the maximum stress in specimens of the G1 group indicates that increasing the volume fraction of SMA fibers in composites made of only SMA fibers (pure composites) significantly increases the maximum stress in the composites. As such, in the G1 group specimens, by increasing the volume fraction of SMA fibers from 8.7% to 20.3%, the maximum stress in the composites increases by 81.30%.
- Comparison of the S7 specimen with other composites in the G2 to G6 groups (hybrid composites) indicates that among composites designed to reach the target elastic modulus, the S7 composite, which has no conventional fibers, has the lowest maximum stress. In other words, the presence of each type of conventional fiber in composites designed to reach the target elastic modulus increases their maximum stress compared to pure composites.
- Evaluation and comparison of maximum stress generated in composites of G2 to G6 groups indicate that in hybrid composites made of a specific type of conventional fibers and SMA fibers of which the initial strain is the same, the higher-volume fraction of SMA fibers (in other words, the lower-volume fraction of conventional fibers) causes the composite to tolerate lower stress.
- With evaluation and comparison of maximum stress values in composites of G2 to G6 groups, it is observed that hybrid composites consist of a specific type of conventional and SMA fibers of which the volume fraction is the same, increasing the initial strain in SMA fibers increases the maximum stress that composites can tolerate.
- Considering the maximum stress values in composites of G2 to G6 groups shows that in composites with equal volume fractions of SMA and conventional fibers and the same initial strain in SMA fibers, the higher failure strain of the conventional fibers used in the composite, the higher maximum stress that can be tolerated by composites. Therefore, hybrid composites produced from high-elastic-modulus carbon (HM-Carbon) fibers have the lowest maximum stress, and hybrid composites made of S-Glass fibers have the highest maximum stress.
- Investigations show that the effect of SMA fibers number on the maximum stress of hybrid composites made of conventional fibers with higher failure strain (composites made of S-Glass and aramid fibers) is greater.

### 4.2. Residual Strain

As mentioned, SMAs are a group of metal alloys that can recover large values of strain during unloading. One of the significant parameters in investigating composites’ behavior is the residual strain of specimens after the unloading.

**Figure 11:**
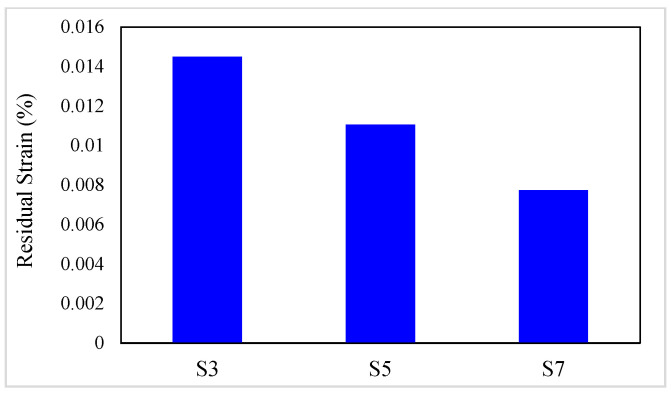
Variation of residual strain in G1 group composites.

**Figure 12:**
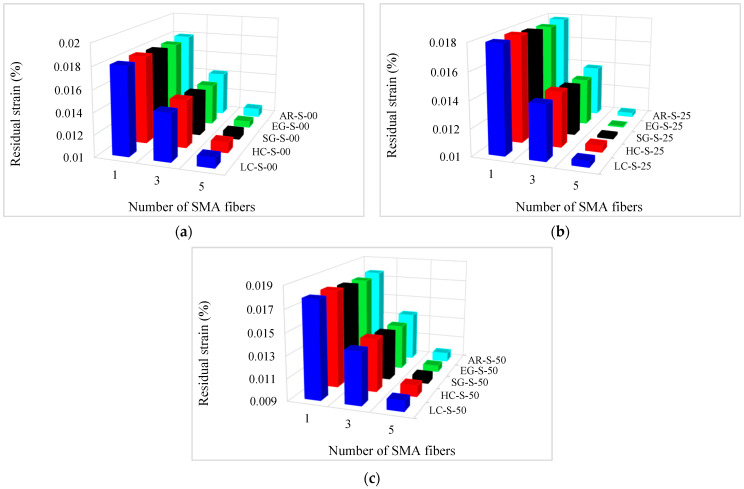
Variation of residual strain in G2 to G6 group composites: (**a**) Pre-strain = 0%; (**b**) Pre-strain = 0.25%; (**c**) Pre-strain = 0.50%.

The residual strain values of composites are presented in Table 6. In addition, the residual strain variation diagrams versus the conventional fiber type, the volume fraction of conventional and SMA fibers, and the initial strain of SMA fibers are presented in Figure 11 and Figure 12. By considering Table 6 and plotted diagrams in Figure 11 and Figure 12, the following results were concluded:

- Considering the residual strain in the G1 group specimens indicates that increasing the volume fraction of SMA fibers in pure composites significantly reduces the residual strain in the composites, so in the G1 group specimens, by increasing the volume fraction of SMA fibers from 8.7% to 20.3%, the residual strain in the composites reduces by 46.62%.
- Comparison of the S7 composite with other composites in the G2 to G6 groups indicates that among composites designed to reach the target elastic modulus, the S7 composite, which has no conventional fibers, has the lowest residual strain. In other words, the presence of each type of conventional fiber in composites designed to reach the target elastic modulus increases their residual strain compared to pure composites.
- Evaluation and comparison of residual strain generated in the G2 to G6 group composites show that in hybrid composites that are made of a specific type of conventional fibers and SMA fibers of which the initial strain is the same, the higher volume fraction of SMA fibers (in other words, the lower volume fraction of conventional fibers) causes less residual strain in composites.
- With evaluation and comparison of residual strain values in composites of G2 to G6 groups, it is observed that in hybrid composites consisting of a specific type of conventional and SMA fibers of which the volume fraction is the same, increasing the initial strain in SMA fibers reduces the residual strain. In addition, investigations show that by increasing the initial strain of SMA fibers from 0 to 0.5%, the residual strain reduction amount depends on the volume fraction of SMA fibers. By increasing the SMA fibers volume fraction in the composites, the effect of the initial strain amount on the residual strain increases. For example, in G2 group composites, by increasing the initial strain of SMA fibers from 0 to 0.5%, the residual strain of composites with 2.9%, 8.7%, and 14.5% SMA fibers reduces 1.1647%, 4.2568%, and 8.9825%, respectively.
- Evaluation and comparison of residual strain values in composites of G2 to G6 groups show that in composites with equal volume fractions of SMA and conventional fibers, and the same initial strain in SMA fibers, the lower the elastic modulus of the conventional fibers used in the composite, the lower the residual strain of the composites. Therefore, hybrid composites produced from high elastic modulus carbon (HM-Carbon) fibers have the highest residual strain, and hybrid composites made of E-Glass fibers have the lowest residual strain.

### 4.3. Energy Dissipation Capability

The energy dissipation was obtained by calculating the area enclosed between the loading and unloading branches of the stress–strain hysteresis curve in the cycles before the conventional fibers rupture [9].

**Figure 13:**
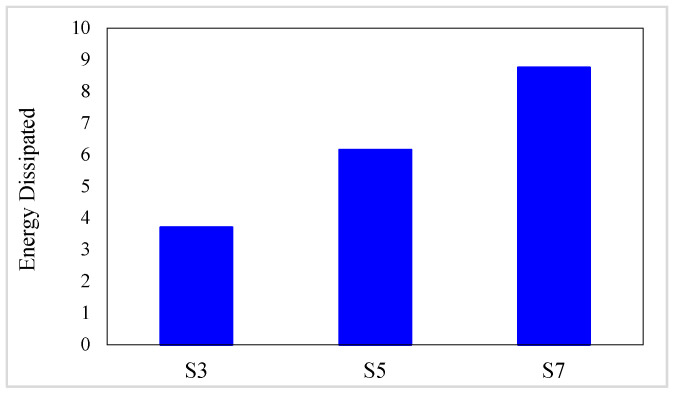
Variation of energy dissipation values in G1 group composites.

**Figure 14:**
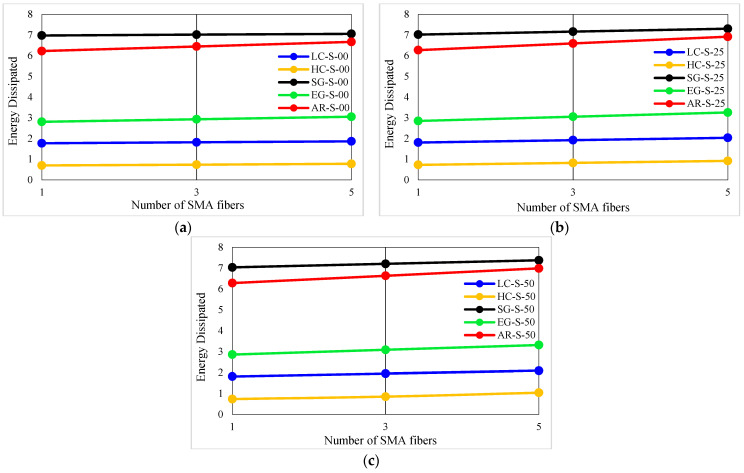
Variation of energy dissipation values in G2 to G6 group composites: (**a**) Pre-strain = 0%; (**b**) Pre-strain = 0.25%; (**c**) Pre-strain = 0.50%.

The energy dissipation values of investigated composites are presented in Table 7. In addition, the energy dissipation variation diagrams versus the conventional fiber type, the volume fraction of conventional and SMA fibers, and the initial strain of SMA fibers are presented in Figure 13 and Figure 14. By considering Table 7 and plotted diagrams in Figure 13 and Figure 14, the following results were concluded:

- Regarding the energy dissipation in the G1 group specimens indicates that increasing the volume fraction of SMA fibers in pure composites significantly increases the energy dissipation in the composites, so in the G1 group composites, by increasing the volume fraction of SMA fibers from 8.7% to 20.3%, the energy dissipation increases by 136.23%.
- Evaluation and comparison of energy dissipation in G2 to G6 group composites demonstrate that in composites made of a specific type of conventional fibers and SMA fibers of which the initial strain is the same, the higher volume fraction of SMA fibers (in other words, the lower volume fraction of conventional fibers) increases the energy dissipation of the composites.
- With evaluation and comparison of energy dissipation values in G2 to G6 group composites, it is observed that in hybrid composites made of a specific type of conventional and SMA fibers of which the volume fraction is the same, increasing the initial strain of SMA fibers increases the energy dissipation values in the composites. In addition, investigations show that by increasing the initial strain of SMA fibers from 0 to 0.5%, the increase in energy dissipation values depends on the volume fraction of SMA fibers. The higher amount of SMA fibers used in the composite, the greater effect of the initial strain on energy dissipation. For example, in G2 group specimens, by increasing the initial strain of SMA fibers from 0 to 0.5%, the energy dissipation of composites with 2.9%, 8.7%, and 14.5%, SMA fibers increases 2.6746%, 7.5570%, and 12.2609%, respectively.
- Evaluation and comparison of energy dissipation values in composites of G2 to G6 groups show that in composites with equal volume fractions of SMA and conventional fibers and the same initial strain in SMA fibers, the higher the failure strain of the conventional fibers used in the composite, the higher the energy dissipation by composites. Therefore, hybrid composites produced from S-Glass fibers have the highest energy dissipation capability, and hybrid composites made of high modulus carbon (HM-Carbon) fibers have the lowest energy dissipation.

### 4.4. Yield Stress

The behavior of SMA-FRP composites until the SMA fibers strain reaches the austenitic-to-martensitic phase transformation strain is linear. After this point, since SMA fibers start the phase transformation, the elastic modulus of SMA reduces, and the stiffness of the investigated composite is decreased. This point is known as the yield point of the SMA-FRP composite, and the stress at this point is equivalent to the composite’s yield stress [60].

**Figure 15:**
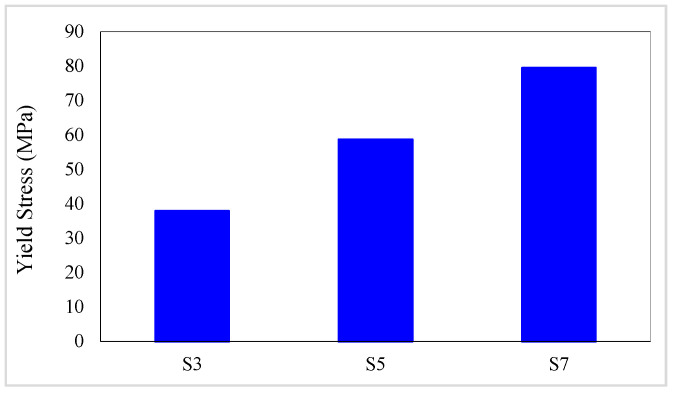
Variation of yield stress in G1 group composites.

**Figure 16:**
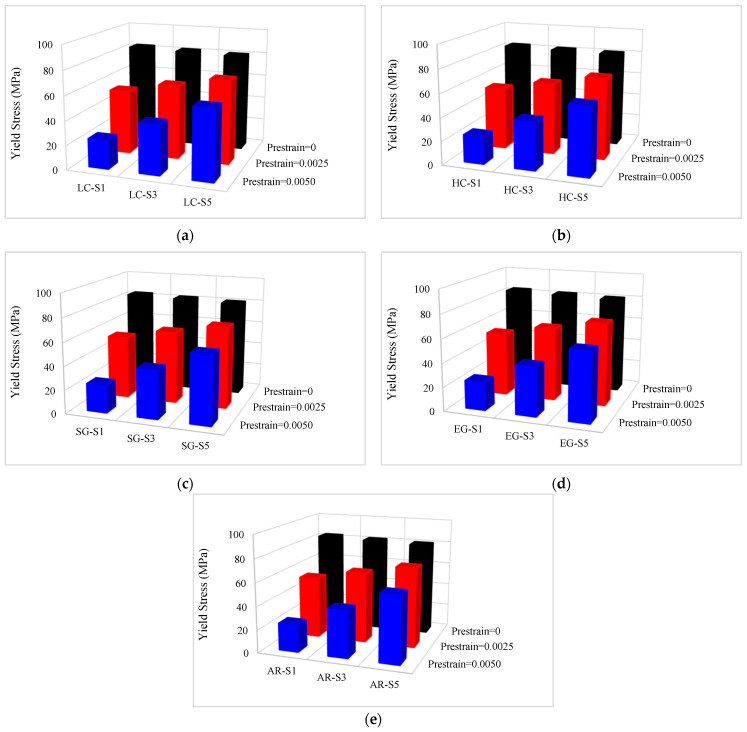
Variation of yield stress in G2 to G6 group composites: (**a**) G2 composites; (**b**) G3 composites; (**c**) G4 composites; (**d**) G5 composites; (**e**) G6 composites.

The yield stress values of composites are presented in Table 8. In addition, the yield stress variation diagrams versus the conventional fiber type, the volume fraction of conventional and SMA fibers, and the initial strain of SMA fibers are presented in Figure 15 and Figure 16. By considering Table 8 and plotted diagrams in Figure 15 and Figure 16, the following results were concluded:

- Assessing the yield stress in the G1 group composites indicates that increasing the volume fraction of SMA fibers in pure composites significantly increases the yield stress in the specimens, so in the G1 group composites, by increasing the volume fraction of SMA fibers from 8.7% to 20.3%, the yield stress in the composite increases by 109.73%.
- Comparison of the S7 composite with the G2 to G6 group composites indicates that among composites designed to reach the target elastic modulus and their SMA fibers have no initial strain, the S7 composite which is a pure composite has the lowest yield stress. However, comparing the yield stress of the S7 composite with G2 to G6 group composites in which SMA fibers have initial strain shows that the yield stress of the S7 composite is higher than all these composites.
- Evaluation and comparison of yield stress in composites of G2 to G6 groups reveal that composites consisting of a specific type of conventional fibers and SMA fibers, if the SMA fibers have no initial strain, increasing the volume fraction of the SMA fibers reduces the yield stress. However, if SMA fibers have initial strain (0.25% and 0.50%), increasing the volume fraction of SMA fibers increases the yield stress.
- With evaluation and comparison of yield stress values in the G2 to G6 group composites, it is observed that in composites made of a specific type of conventional and SMA fibers of which the volume fraction is the same, increasing the initial strain in SMA fibers decreases the yield stress. In addition, the investigations show that yield stress reduction value by increasing the initial strain of SMA fibers depends on the volume fraction of SMA fibers. For example, in the G2 group, by increasing the initial strain of SMA fibers from 0 to 0.5%, the yield stress of composites with 2.9%, 8.7%, and 14.5% of SMA fibers decreases 71.37%, 50.09%, and 33.60%, respectively. In other words, with increasing the volume fraction of SMA fibers, the effect of the initial strain on the yield stress of the composite decreases.
- Evaluation and comparison of yield stress values in the G2 to G6 group composites show that in composites with equal volume fractions of SMA and conventional fibers and the same initial strain created in SMA fibers, changing the type of conventional fibers used in hybrid composite construction has a very small effect on yield stress value (approximately 0.39% on average). Therefore, it can be said that the amount of yield stress of the hybrid composites is independent of the type of conventional fibers used in producing the composite.

### 4.5. Residual Stress

One significant characteristic of SMA-FRP composites is their ability to maintain a reasonable percentage of their load-carrying capacity after the failure of conventional fibers. This characteristic is very important, especially when the structure is under very large loads. Under such loading conditions, the acceptable residual stress in the SMA-FRP composites can protect the structure against collapse [60].

**Figure 17:**
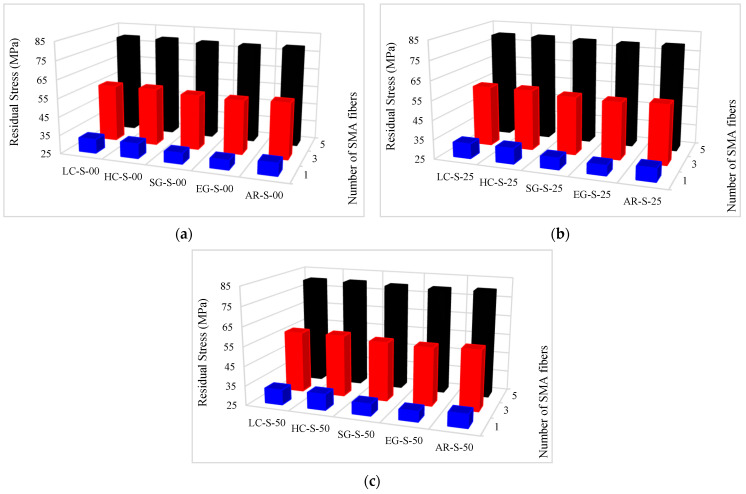
Variation of residual stress values in G2 to G6 group composites: (**a**) Pre-strain = 0%; (**b**) Pre-strain = 0.25%; (**c**) Pre-strain = 0.50%.

The residual stress values of composites are presented in Table 9. In addition, the residual stress variation diagrams versus the conventional fiber type, the volume fraction of conventional and SMA fibers, and the initial strain of SMA fibers are presented in Figure 17. By considering Table 9 and plotted diagrams in Figure 17, the following results were concluded:

- Considering residual stress in the G2 to G6 group composites reveals that in composites made of a specific type of conventional fibers and SMA fibers and where the initial strain of SMA fibers is the same, increasing the volume fraction of SMA fibers (in other words, decreasing the volume fraction of conventional fibers) significantly increases the residual stress of the composites. For example, in G2 group composites, with increasing the volume fraction of SMA fibers used in composites from 2.9% to 14.5%, the amount of residual stress in the composites increases by 144.39% on average.
- With evaluation and comparison of residual stress values in the G2 to G6 group composites, it is observed that in hybrid composites made of a specific type of conventional and SMA fibers of which the volume fraction is the same, increasing the initial strain in SMA fibers increases the residual stress. In addition, the investigations show that by increasing the initial strain of SMA fibers, increasing the residual stress value depends on the volume fraction of SMA fibers. For example, in the G2 group composites, by increasing the initial strain of SMA fibers from 0 to 0.5%, the residual stress of composites with 2.9%, 8.7%, and 14.5% of SMA fibers increases 0.68%, 1.19%, and 1.41%, respectively. In other words, by increasing the volume fraction of SMA fibers, the effect of the initial strain on the residual stress value increases. On the other hand, considering the very small change of residual stress with changing the initial strain applied to the SMA fibers used in the composite, it can be concluded with high accuracy that the value of the residual stress in the SMA-FRP composite is independent of the initial strain in the SMA fibers.
- Evaluation and comparison of residual stress values in the G2 to G6 group composites show that in hybrid composites with equal volume fractions of SMA and conventional fibers and the same initial strain in SMA fibers, the higher the elastic modulus of the conventional fibers used in producing the composites, the higher the residual stress in composites. Therefore, composites produced from high modulus carbon (HM-Carbon) fibers have the highest residual stress, and the composites made of E-Glass fibers have the lowest residual stress. The reason is that after the failure of conventional fibers, load bearing in the composite is the responsibility of SMA fibers and resin. By increasing the elastic modulus of conventional fibers, due to the constant volume fraction of SMA fibers, the required volume fraction of conventional fibers will be lower, and the required resin volume fraction will be higher (as stated, the design of composites to reach a target elastic modulus is done).

### 4.6. Strain Hardening Ratio

As mentioned in previous sections, until the strain of SMA fibers reaches the austenitic-to-martensitic phase transformation strain, the behavior of the SMA-FRP composite is linear. After this point, due to the SMA fiber phase transformation, the elastic modulus of SMA is reduced and, as a result, the composite stiffness decreases. This point is known as the yield point of the SMA-FRP composite. The stiffness of the SMA-FRP composites is significant after the yield point. The ratio of composite stiffness after the yield point to the initial composite stiffness is named the strain hardening ratio. The strain hardening ratio of the composite defines the ratio of composite stiffness drop after reaching the yield point [60].

**Figure 18:**
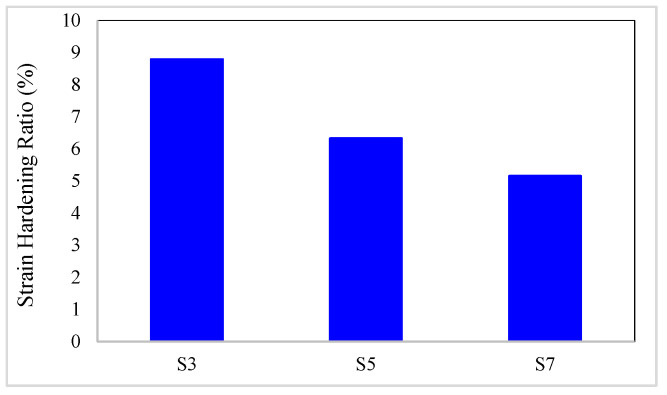
Variation of strain hardening ratio in G1 group composites.

**Figure 19:**
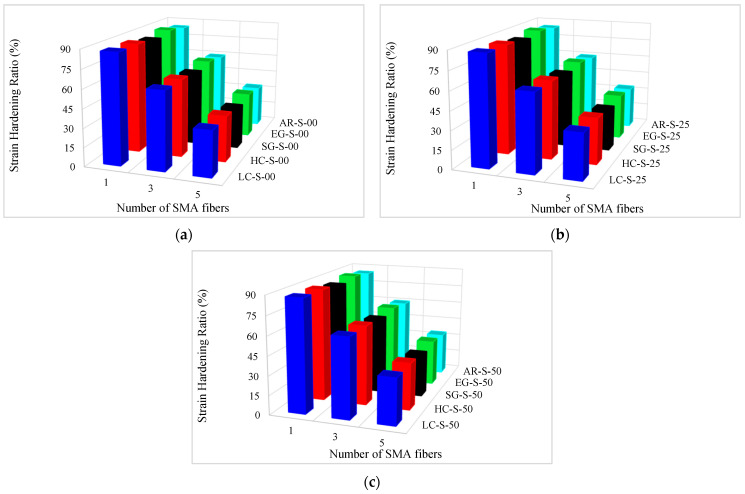
Variation of strain hardening ratio in G2 to G6 group composites: (**a**) pre-strain = 0%; (**b**) pre-strain = 0.25%; (**c**) pre-strain = 0.50%.

The strain hardening ratio of composites is presented in Table 10. In addition, the strain hardening ratio variation diagrams versus the conventional fiber type, the volume fraction of conventional and SMA fibers, and the initial strain of SMA fibers are presented in Figure 18 and Figure 19. By considering Table 10 and plotted diagrams in Figure 18 and Figure 19, the following results were concluded:

- Regarding the strain hardening ratio in the G1 group specimens indicates that increasing the volume fraction of SMA fibers in pure composites significantly decreases the strain hardening ratio in the composites. As such, in the G1 group specimens, with increasing the volume fraction of SMA fibers from 8.7% to 20.3%, the strain hardening ratio in the composite decreases by 41.29%.
- Comparison of the S7 specimen with the G2 to G6 group composites indicates that among composites designed to reach the target elastic modulus, the S7 composite, which is a pure composite, has the lowest strain hardening ratio. In other words, the presence of each type of conventional fiber in hybrid composites designed to reach the target elastic modulus increases their strain hardening ratio compared to pure composites.
- Evaluation and comparison of strain hardening ratio in the G2 to G6 group composites reveal that in composites made of a specific type of conventional fibers and SMA fibers of which the initial strain percentage is the same, the higher volume fraction of SMA fibers (in other words, the lower volume fraction of conventional fibers) decreases strain hardening ratio. For example, in G2 group composites, with an increase in the volume fraction of SMA fibers from 2.9% to 14.5%, the strain hardening ratio decreases by 58.87% on average.
- With evaluation and comparison of strain hardening ratio in the G2 to G6 group composites, it is observed that in hybrid composites made of a specific type of conventional and SMA fibers of which the volume fraction is the same, by changing the initial strain percentage in SMA fibers, the strain hardening ratio of composites does not change (the changes are very small). Therefore, it can be stated that the strain hardening ratio of SMA-FRP composites is independent of the initial strain percentage in SMA fibers.
- Evaluation and comparison of strain hardening ratio in the G2 to G6 group composites show that in composites with equal volume fractions of SMA and conventional fibers and the same initial strain in SMA fibers, the higher the failure strain of the conventional fibers used in composites, the smaller the strain hardening ratio in composites. Therefore, composites produced from high elastic modulus carbon (HM-Carbon) fibers have the highest strain hardening ratio, and the composites made of S-Glass fibers have the lowest strain hardening ratio. The investigations show that the amount of these changes is minor.

In the end, to summarize the results of the investigations, the effect of the volume fraction of SMA fibers, the effect of the initial strain percentage in SMA fibers, and also the effect of conventional fibers type used in the producing hybrid composites on the six investigated parameters are presented in Table 11, Table 12 and Table 13. In these tables, the meaning of direct ratio is that with increasing the studied variable, the desired parameter increases, and with decreasing the variable, the desired parameter decreases. The meaning of the inverse ratio is that with increasing the studied variable, the desired parameter decreases, and with decreasing the variable, the desired parameter increases. In addition, the meaning of independent is that it can be said with high accuracy that the value of the desired parameter does not change with varying the investigated variable value.

## 5. Conclusions

This study investigates the effect of using different volume fractions of conventional fibers (carbon, glass, and aramid) and SMA fibers (NiTi) in the super-elastic phase and the effect of the initial strain percentage of SMA fibers on the SMA-FRP composites’ behavior under cyclic tensile loading. Analyzing the results shows the following:

- In composites made of only SMA fibers (pure composites), increasing the volume fraction of SMA fibers increases the maximum stress, energy dissipation capability, and yield stress, and also reduces the residual strain and strain hardening ratio of the composite.
- By comparing the pure composites and hybrid composites that are designed to reach a target elastic modulus, it can be seen that the maximum stress, residual strain, and strain hardening ratio of pure composites are lower than those of the hybrid composites. In other words, the presence of each type of conventional fiber in hybrid composites designed to reach the target elastic modulus increases their maximum stress, residual strain, and strain hardening ratio compared to pure composites.
- By comparing pure composites and hybrid composites that are designed to reach a target elastic modulus and their SMA fibers having no initial strain, the pure composites have the lowest yield stress. However, comparing the yield stress of the pure composites with hybrid composites in which the initial strain of SMA fibers is not zero, shows that the yield stress of the pure composites is higher than all hybrid composites.
- In hybrid composites that are designed to achieve a target elastic modulus, with an increase in the volume fraction of SMA fibers, the maximum stress, residual strain, and strain hardening ratio decrease, and the energy dissipation capability and residual stress of the composites increase.
- In hybrid composites that are designed to achieve a target elastic modulus, increasing the volume fraction of SMA fibers with no initial strain reduces the yield stress of the composite. However, increasing the volume fraction of SMA fibers with an initial strain (0.25% and 0.50% in this research) will increase the yield stress of the composite.
- In hybrid composites that are designed to achieve a target elastic modulus, increasing the initial strain percentage in SMA fibers increases the maximum stress and energy dissipation capability and reduces the residual strain and yield stress.
- In hybrid composites that are designed to achieve a specific elastic modulus, the amount of residual stress and the strain hardening ratio of the composites are independent of the initial strain percentage in the SMA fibers.
- In hybrid composites that are designed to achieve a specific elastic modulus, fibers with a larger failure strain increase the maximum stress and energy dissipation capability of the composites and reduce the strain hardening ratio of the composites.
- In hybrid composites that are designed to achieve a specific elastic modulus, increasing the elastic modulus of conventional fibers increases the residual strain and residual stress of the composites.
- In hybrid composites that are designed to achieve a target elastic modulus, the yield stress in the composites is independent of the conventional fibers type used in producing the composites.

## Figures and Tables

**Table 1:** Mechanical properties of materials.

Number	Material	Mechanical Properties	Abvn.	Value
1	Resin [14]	Young’s Modulus	E_m_	E_m_ = 1.57 GPa
		Yield Stress	F_ym_	F_ym_ = 22 MPa
2	Nitinol fibers [14]	Young’s Modulus	E_SMA_	E_SMA_ = 63.50 GPa
		Austenite to Martensite start stress	σ_AMS_	σ_AMS_ = 365 MPa
		Austenite to Martensite finish stress	σ_AMf_	σ_AMf_ = 425 MPa
		Martensite to Austenite start stress	σ_MAS_	σ_MAS_ = 102 MPa
		Martensite to Austenite finish stress	σ_MAf_	σ_MAf_ = 50 MPa
3	Low Modulus carbon fiber [67]	Young’s Modulus	E_f_	E_f_ = 230 GPa
		Rupture Strain	$\varepsilon_{f}$	ε = 1.6%
4	High Modulus carbon fiber [67]	Young’s Modulus	E_f_	E_f_ = 370 GPa
		Rupture Strain	$\varepsilon_{f}$	ε = 1%
5	S-Glass fiber [14]	Young’s Modulus	E_f_	E_f_ = 86.7 GPa
		Rupture Strain	$\varepsilon_{f}$	ε = 3.20%
6	E-Glass fiber [67]	Young’s Modulus	E_f_	E_f_ = 68.9 GPa
		Rupture Strain	$\varepsilon_{f}$	ε = 2%
7	Aramid fiber [67]	Young’s Modulus	E_f_	E_f_ = 124.1 GPa
		Rupture Strain	$\varepsilon_{f}$	ε = 3%

**Table 2:** Introduction of the investigated groups.

Number	Group Name	Description
1	G1	composites made of nitinol fibers (pure composites)
2	G2	composites made of a combination of nitinol fibers and low-modulus carbon (LM-Carbon) fibers (hybrid composites)
3	G3	composites made of a combination of nitinol fibers and high-modulus carbon (HM-Carbon) fibers (hybrid composites)
4	G4	composites made of a combination of nitinol fibers and S-Glass fibers (hybrid composites)
5	G5	composites made of a combination of nitinol fibers and E-Glass fibers (hybrid composites)
6	G6	composites made of a combination of nitinol fibers and Aramid fibers (hybrid composites)

**Table 3:** Introduction of SMA-FRP composites.

Group Name	Specimen Name	N_SMA_	A_SMA_ (mm^2^)	V_SMA_	SMA Pre-strain (%)	V_f_	V_m_	E_c_ (GPa)
**G1**	S3	3	0.5889	0.087	0	0	0.913	6.9579
	S5	5	0.9815	0.145	0	0	0.855	10.5498
	S7	7	1.3741	0.203	0	0	0.797	14.1418
**G2**	LC-S1-00	1	0.1963	0.029	0.00	0.0471	0.9239	14.1022
	LC-S1-25	1	0.1963	0.029	0.25	0.0471	0.9239	14.1022
	LC-S1-50	1	0.1963	0.029	0.50	0.0471	0.9239	14.1022
	LC-S3-00	3	0.5889	0.087	0.00	0.0314	0.8816	14.1306
	LC-S3-25	3	0.5889	0.087	0.25	0.0314	0.8816	14.1306
	LC-S3-50	3	0.5889	0.087	0.50	0.0314	0.8816	14.1306
	LC-S5-00	5	0.9815	0.145	0.00	0.0157	0.8393	14.1362
	LC-S5-25	5	0.9815	0.145	0.25	0.0157	0.8393	14.1362
	LC-S5-50	5	0.9815	0.145	0.50	0.0157	0.8393	14.1362
**G3**	HC-S1-00	1	0.1963	0.029	0.00	0.0292	0.9418	14.1241
	HC-S1-25	1	0.1963	0.029	0.25	0.0292	0.9418	14.1241
	HC-S1-50	1	0.1963	0.029	0.50	0.0292	0.9418	14.1241
	HC-S3-00	3	0.5889	0.087	0.00	0.0195	0.8935	14.1423
	HC-S3-25	3	0.5889	0.087	0.25	0.0195	0.8935	14.1423
	HC-S3-50	3	0.5889	0.087	0.50	0.0195	0.8935	14.1423
	HC-S5-00	5	0.9815	0.145	0.00	0.0100	0.8450	14.2341
	HC-S5-25	5	0.9815	0.145	0.25	0.0100	0.8450	14.2341
	HC-S5-50	5	0.9815	0.145	0.50	0.0100	0.8450	14.2341
**G4**	SG-S1-00	1	0.1963	0.029	0.00	0.1265	0.8445	14.1349
	SG-S1-25	1	0.1963	0.029	0.25	0.1265	0.8445	14.1349
	SG-S1-50	1	0.1963	0.029	0.50	0.1265	0.8445	14.1349
	SG-S3-00	3	0.5889	0.087	0.00	0.0844	0.8286	14.1428
	SG-S3-25	3	0.5889	0.087	0.25	0.0844	0.8286	14.1428
	SG-S3-50	3	0.5889	0.087	0.50	0.0844	0.8286	14.1428
	SG-S5-00	5	0.9815	0.145	0.00	0.0422	0.8128	14.1423
	SG-S5-25	5	0.9815	0.145	0.25	0.0422	0.8128	14.1423
	SG-S5-50	5	0.9815	0.145	0.50	0.0422	0.8128	14.1423
**G5**	EG-S1-00	1	0.1963	0.029	0.00	0.1600	0.8110	14.1388
	EG-S1-25	1	0.1963	0.029	0.25	0.1600	0.8110	14.1388
	EG-S1-50	1	0.1963	0.029	0.50	0.1600	0.8110	14.1388
	EG-S3-00	3	0.5889	0.087	0.00	0.1067	0.8063	14.1420
	EG-S3-25	3	0.5889	0.087	0.25	0.1067	0.8063	14.1420
	EG-S3-50	3	0.5889	0.087	0.50	0.1067	0.8063	14.1420
	EG-S5-00	5	0.9815	0.145	0.00	0.0533	0.8017	14.1385
	EG-S5-25	5	0.9815	0.145	0.25	0.0533	0.8017	14.1385
	EG-S5-50	5	0.9815	0.145	0.50	0.0533	0.8017	14.1385
**G6**	AR-S1-00	1	0.1963	0.029	0.00	0.0879	0.8831	14.1363
	AR-S1-25	1	0.1963	0.029	0.25	0.0879	0.8831	14.1363
	AR-S1-50	1	0.1963	0.029	0.50	0.0879	0.8831	14.1363
	AR-S3-00	3	0.5889	0.087	0.00	0.0586	0.8544	14.1381
	AR-S3-25	3	0.5889	0.087	0.25	0.0586	0.8544	14.1381
	AR-S3-50	3	0.5889	0.087	0.50	0.0586	0.8544	14.1381
	AR-S5-00	5	0.9815	0.145	0.00	0.0293	0.8257	14.1400
	AR-S5-25	5	0.9815	0.145	0.25	0.0293	0.8257	14.1400
	AR-S5-50	5	0.9815	0.145	0.50	0.0293	0.8257	14.1400

N_SMA_ = Number of SMA Fibers; A_SMA_ = SMA Fiber Cross Section Area.

**Table 4:** Characteristics of the experimental specimen used to calibrate the numerical simulation [14].

Specimen Name	Volume Fraction of SMA Fiber	Volume Fraction of S-Glass Fiber	Volume Fraction of Resin	Elastic Modulus of SMA	Elastic Modulus of S-Glass	Elastic Modulus of Resin
PRC3	0.08	0.09	0.83	60.12 Gpa	86.70 Gpa	1.57 Gpa

**Table 5:** Maximum stress values in investigated composites (MPa).

Specimen Name	Maximum Stress	Specimen Name	Maximum Stress	Specimen Name	Maximum Stress	Specimen Name	Maximum Stress
S3	56.382	HC-S1-00	129.032	SG-S3-00	287.540	EG-S5-00	147.053
S5	79.304	HC-S1-25	129.251	SG-S3-25	287.937	EG-S5-25	147.648
S7	102.223	HC-S1-50	129.352	SG-S3-50	288.260	EG-S5-50	148.195
LC-S1-00	200.483	HC-S3-00	114.167	SG-S5-00	193.632	AR-S1-00	358.223
LC-S1-25	200.705	HC-S3-25	114.553	SG-S5-25	194.219	AR-S1-25	358.335
LC-S1-50	200.809	HC-S3-50	114.856	SG-S5-50	194.768	AR-S1-50	358.447
LC-S3-00	164.055	HC-S5-00	100.040	EG-S1-00	249.393	AR-S3-00	271.848
LC-S3-25	164.437	HC-S5-25	100.631	EG-S1-25	249.515	AR-S3-25	272.236
LC-S3-50	164.736	HC-S5-50	101.175	EG-S1-50	249.637	AR-S3-50	272.515
LC-S5-00	127.627	SG-S1-00	381.172	EG-S3-00	198.291	AR-S5-00	185.474
LC-S5-25	128.210	SG-S1-25	381.289	EG-S3-25	198.702	AR-S5-25	186.057
LC-S5-50	128.745	SG-S1-50	381.405	EG-S3-50	199.029	AR-S5-50	186.601

**Table 6:** Residual strain values in investigated composites.

Specimen Name	Residual Strain	Specimen Name	Residual Strain	Specimen Name	Residual Strain	Specimen Name	Residual Strain
S3	0.014	HC-S1-00	0.01806	SG-S3-00	0.014	EG-S5-00	0.010
S5	0.011	HC-S1-25	0.01795	SG-S3-25	0.013	EG-S5-25	0.010
S7	0.007	HC-S1-50	0.01785	SG-S3-50	0.013	EG-S5-50	0.0095
LC-S1-00	0.018	HC-S3-00	0.01440	SG-S5-00	0.010	AR-S1-00	0.017
LC-S1-25	0.017	HC-S3-25	0.01409	SG-S5-25	0.010	AR-S1-25	0.017
LC-S1-50	0.017	HC-S3-50	0.01379	SG-S5-50	0.0096	AR-S1-50	0.017
LC-S3-00	0.014	HC-S5-00	0.01096	EG-S1-00	0.017	AR-S3-00	0.014
LC-S3-25	0.014	HC-S5-25	0.01047	EG-S1-25	0.017	AR-S3-25	0.013
LC-S3-50	0.013	HC-S5-50	0.009994	EG-S1-50	0.017	AR-S3-50	0.013
LC-S5-00	0.010	SG-S1-00	0.01785	EG-S3-00	0.013	AR-S5-00	0.010
LC-S5-25	0.010	SG-S1-25	0.01773	EG-S3-25	0.013	AR-S5-25	0.010
LC-S5-50	0.009	SG-S1-50	0.01761	EG-S3-50	0.013	AR-S5-50	0.009

**Table 7:** Energy Dissipation values in investigated composites.

Specimen Name	Energy Dissipated	Specimen Name	Energy Dissipated	Specimen Name	Energy Dissipated	Specimen Name	Energy Dissipated
S3	3.703	HC-S1-00	0.700	SG-S3-00	7.020	EG-S5-00	3.054
S5	6.151	HC-S1-25	0.729	SG-S3-25	7.166	EG-S5-25	3.259
S7	8.748	HC-S1-50	0.740	SG-S3-50	7.208	EG-S5-50	3.319
LC-S1-00	1.772	HC-S3-00	0.738	SG-S5-00	7.058	AR-S1-00	6.228
LC-S1-25	1.808	HC-S3-25	0.822	SG-S5-25	7.306	AR-S1-25	6.273
LC-S1-50	1.819	HC-S3-50	0.855	SG-S5-50	7.378	AR-S1-50	6.287
LC-S3-00	1.819	HC-S5-00	0.779	EG-S1-00	2.809	AR-S3-00	6.449
LC-S3-25	1.922	HC-S5-25	0.919	EG-S1-25	2.850	AR-S3-25	6.595
LC-S3-50	1.957	HC-S5-50	1.045	EG-S1-50	2.862	AR-S3-50	6.636
LC-S5-00	1.866	SG-S1-00	6.979	EG-S3-00	2.932	AR-S5-00	6.670
LC-S5-25	2.037	SG-S1-25	7.024	EG-S3-25	3.056	AR-S5-25	6.920
LC-S5-50	2.095	SG-S1-50	7.038	EG-S3-50	3.092	AR-S5-50	6.991

**Table 8:** Yield stress values in investigated composites (MPa).

Specimen Name	Yield Stress	Specimen Name	Yield Stress	Specimen Name	Yield Stress	Specimen Name	Yield Stress
S3	37.879	HC-S1-00	82.804	SG-S3-00	81.950	EG-S5-00	80.707
S5	58.663	HC-S1-25	53.314	SG-S3-25	61.450	EG-S5-25	69.361
S7	79.447	HC-S1-50	23.706	SG-S3-50	40.866	EG-S5-50	57.966
LC-S1-00	82.861	HC-S3-00	81.760	SG-S5-00	80.698	AR-S1-00	83.047
LC-S1-25	53.351	HC-S3-25	61.319	SG-S5-25	69.355	AR-S1-25	53.350
LC-S1-50	23.722	HC-S3-50	40.794	SG-S5-50	57.963	AR-S1-50	23.653
LC-S3-00	81.723	HC-S5-00	81.166	EG-S1-00	83.269	AR-S3-00	81.847
LC-S3-25	61.293	HC-S5-25	69.628	EG-S1-25	53.491	AR-S3-25	61.379
LC-S3-50	40.780	HC-S5-50	58.042	EG-S1-50	23.714	AR-S3-50	40.827
LC-S5-00	80.585	SG-S1-00	83.149	EG-S3-00	82.009	AR-S5-00	80.647
LC-S5-25	69.276	SG-S1-25	53.415	EG-S3-25	61.491	AR-S5-25	69.319
LC-S5-50	57.921	SG-S1-50	23.681	EG-S3-50	40.889	AR-S5-50	57.944

**Table 9:** Residual stress values in investigated composites (MPa).

Specimen Name	Residual Stress	Specimen Name	Residual Stress	Specimen Name	Residual Stress	Specimen Name	Residual Stress
S3	-------	HC-S1-00	32.818	SG-S3-00	54.525	EG-S5-00	78.131
S5	-------	HC-S1-25	32.929	SG-S3-25	54.860	EG-S5-25	78.688
S7	-------	HC-S1-50	33.041	SG-S3-50	55.194	EG-S5-50	79.246
LC-S1-00	32.424	HC-S3-00	55.953	SG-S5-00	78.375	AR-S1-00	31.527
LC-S1-25	32.536	HC-S3-25	56.287	SG-S5-25	78.932	AR-S1-25	31.638
LC-S1-50	32.647	HC-S3-50	56.621	SG-S5-50	79.490	AR-S1-50	31.749
LC-S3-00	55.691	HC-S5-00	79.084	EG-S1-00	29.940	AR-S3-00	55.093
LC-S3-25	56.025	HC-S5-25	79.641	EG-S1-25	30.052	AR-S3-25	55.427
LC-S3-50	56.359	HC-S5-50	80.198	EG-S1-50	30.163	AR-S3-50	55.761
LC-S5-00	78.958	SG-S1-00	30.677	EG-S3-00	54.035	AR-S5-00	78.659
LC-S5-25	79.515	SG-S1-25	30.788	EG-S3-25	54.369	AR-S5-25	79.216
LC-S5-50	80.072	SG-S1-50	30.900	EG-S3-50	54.704	AR-S5-50	79.773

**Table 10:** Strain hardening ratio in investigated composites.

Specimen Name	Strain Hardening Ratio (%)	Specimen Name	Strain Hardening Ratio (%)	Specimen Name	Strain Hardening Ratio (%)	Specimen Name	Strain Hardening Ratio (%)
S3	8.7896	HC-S1-00	87.3200	SG-S3-00	59.0857	EG-S5-00	36.0758
S5	6.3313	HC-S1-25	87.5851	SG-S3-25	59.4562	EG-S5-25	36.1493
S7	5.1586	HC-S1-50	87.7073	SG-S3-50	59.7667	EG-S5-50	36.1658
LC-S1-00	87.4643	HC-S3-00	61.9953	SG-S5-00	32.9600	AR-S1-00	84.5696
LC-S1-25	87.6026	HC-S3-25	62.1867	SG-S5-25	33.2905	AR-S1-25	84.8828
LC-S1-50	87.7290	HC-S3-50	62.2319	SG-S5-50	33.5374	AR-S1-50	85.2181
LC-S3-00	62.0752	HC-S5-00	36.3707	EG-S1-00	87.5509	AR-S3-00	49.2492
LC-S3-25	62.1832	HC-S5-25	36.4953	EG-S1-25	87.5965	AR-S3-25	59.6139
LC-S3-50	62.2257	HC-S5-50	36.5047	EG-S1-50	87.7167	AR-S3-50	59.8989
LC-S5-00	35.9689	SG-S1-00	84.4147	EG-S3-00	62.2251	AR-S5-00	33.1755
LC-S5-25	36.0328	SG-S1-25	84.7252	EG-S3-25	62.3508	AR-S5-25	33.4982
LC-S5-50	36.0283	SG-S1-50	85.0631	EG-S3-50	62.4405	AR-S5-50	33.7319

**Table 11:** Investigating the effect of the volume fraction of SMA fibers on the investigated composites’ behavior (assuming the constant initial strain percentage in SMA fibers and the specific type of conventional fibers in the compared composites).

Composite Type	Maximum Stress	Residual Strain	Energy Dissipation	Yield Stress	Residual Stress	Strain Hardening Ratio
**Pure Composite**	direct ratio	inverse ratio	direct ratio	direct ratio	#N/A	inverse ratio
**Hybrid Composite**	inverse ratio	inverse ratio	direct ratio	If the initial strain of SMA fibers is equal to zero, it has an inverse ratio and if the initial strain of SMA fibers is not zero, the ratio is direct.	direct ratio	inverse ratio

#N/A: Not Available.

**Table 12:** Investigating the effect of the initial strain percentage in SMA fibers on the investigated composites’ behavior (assuming the constant volume fraction of SMA fibers and the specific type of conventional fibers in the compared composites).

Composite Type	Maximum Stress	Residual Strain	Energy Dissipation	Yield Stress	Residual Stress	Strain Hardening Ratio
**Hybrid Composite**	direct ratio	inverse ratio	direct ratio	inverse ratio	independent	independent

**Table 13:** Investigating the effect of the conventional fibers type on the investigated composites’ behavior (assuming the constant volume fraction of SMA fibers and the constant initial strain percentage in SMA fibers in the compared composites).

**Composite Type: Hybrid Composite**	**Maximum Stress**	**The Higher the Failure Strain of the Conventional Fibers, the Higher the Maximum Stress that the Composite Can Tolerate.**
	**Residual strain**	The lower the elastic modulus of the conventional fibers, the lower the residual strain in the composite.
	**Energy dissipation**	The higher the failure strain of the conventional fibers, the higher the energy dissipation by the composite.
	**Yield stress**	Independent
	**Residual stress**	The higher the elastic modulus of the conventional fibers, the higher the residual stress in the composite.
	**Stress hardening ratio**	The higher the failure strain of the conventional fibers, the lower the strain hardening ratio in the composite.

## Data Availability

The data presented in this study are available on request from the corresponding author.
